# Single-Camera AI-Based Classification of Clinically Defined Grasp Patterns in Children with Cerebral Palsy

**DOI:** 10.3390/s26216848

**Published:** 2026-10-29

**Authors:** Tae Won Kim, Sun Young Joo, Su Min Kim, In Jin Yoon, June-Goo Lee, Eun Jae Ko

**Affiliations:** 1 Department of Biomedical Engineering, Asan Medical Center, University of Ulsan College of Medicine, Seoul 05505, Republic of Korea; 2 Department of Rehabilitation Medicine, University of Ulsan College of Medicine, Ulsan 44610, Republic of Korea; 3 Department of Rehabilitation Medicine, Asan Institute for Life Sciences, Foundation for Industry Cooperation, University of Ulsan, Seoul 05505, Republic of Korea; 4 Department of Rehabilitation Medicine, Asan Medical Center, Seoul 05505, Republic of Korea; 5 Department of Rehabilitation Medicine, Asan Medical Center, University of Ulsan College of Medicine, Seoul 05505, Republic of Korea

**Keywords:** cerebral palsy, hand function, grasp pattern classification, QUEST assessment, single-camera video analysis, AI-based classification, markerless hand tracking

## Abstract

Camera-based sensing may provide a scalable approach to quantifying hand function in children with cerebral palsy (CP), but fine grasp patterns remain challenging because of occlusion, small anatomical structures, and complex hand–obobject interactions. This study investigated a single-camera AI-based framework for classifying the developmentally ordered grasp patterns of the Quality of Upper Extremity Skills Test (QUEST). Grasp-of-Cereal and Grasp-of-Cube tasks performed by 41 children with CP were recorded with a single RGB camera and analyzed using MediaPipe hand landmarks, SAM2-based hand segmentation, YOLOv9 object localization, and a 3D DenseNet-121 classifier. Trial-level accuracy, severity-weighted accuracy (SWA), and quadratic weighted kappa (QWK) were estimated with participant-level bootstrap 95% confidence intervals (CIs). Accuracy was 0.84 (95% CI, 0.750–0.914) for Cereal and 0.85 (0.795–0.901) for Cube; SWA was 0.922 and 0.907, and QWK was 0.579 and 0.756, respectively. Therapist inter-rater QWK (0.883 and 0.838) is reported as a descriptive reference only. Cereal macro-F1 (0.534) and balanced accuracy (0.494) indicated uneven class performance. Development and test recordings were temporally separated but not participant-independent; for Cube, 39 of the 41 children contributing development data also contributed follow-up test recordings. These findings support the feasibility of longitudinal within-cohort grasp classification but do not establish generalization to previously unseen children.

## 1. Introduction

Quantitative assessment of upper extremity function in children with cerebral palsy (CP) remains challenging, particularly for fine hand movements that are difficult to characterize using conventional clinical observation alone. Instrumented upper-limb assessments commonly quantify predefined kinematic variables such as joint angles, velocity, and smoothness, but these measures may not fully capture finger coordination or the qualitative structure of functional grasping [1]. Camera-based sensing offers a non-contact alternative that can capture clinically relevant movement information without body-worn markers or dedicated hardware.

Hand and finger movements are particularly demanding targets for vision-based sensing because they involve multiple joints, short anatomical segments, rapid coordination, and frequent self-occlusion [2]. Marker-based optical motion-capture systems can provide three-dimensional kinematic information, including joint trajectories and joint-angle estimates, and remain an important quantitative reference. However, hand analysis requires accurate placement and tracking of markers on small anatomical segments, together with dedicated equipment and setup time, which can be burdensome in pediatric rehabilitation. Single-camera RGB sensing combined with markerless hand tracking could provide a more scalable approach, but hand tracking remains substantially more challenging than whole-body tracking because of occlusion, small structures, and rapid finger motion; its ability to distinguish clinically meaningful grasp patterns in children with CP therefore remains insufficiently established [3,4].

A single frontal camera was selected to minimize acquisition burden and maintain a clinically practical setup that can be reproduced without multi-camera calibration or body-worn sensors. This design trades ease of deployment for intrinsic limitations of monocular imaging, particularly loss of depth information, viewpoint dependence, and hand–object self-occlusion; these limitations are considered explicitly in the interpretation of the present findings.

Recent advances in markerless computer vision have improved the feasibility of extracting hand and finger information from standard RGB video. In healthy adults, MediaPipe-based pose estimation has shown lower joint-angle estimation errors than Leap Motion Controller-based systems across several hand-movement tasks, supporting the potential of camera-based markerless sensing for hand analysis [2]. However, technical performance in healthy participants does not establish clinical validity in children with CP, whose movements may be slower, atypical, variable, or affected by occlusion and impaired selective motor control.

Recent upper-limb validation work has compared markerless estimates with optoelectronic reference measurements across full and partial ranges of motion, highlighting the influence of viewpoint and occlusion on agreement [5]. Reviews of upper-limb assessment in stroke rehabilitation and markerless motion capture in industrial ergonomics likewise emphasize the dependence of validity on task, acquisition conditions, and validation protocols [6,7]. These findings support further investigation of practical camera-based acquisition, but do not establish validity for fine-grasp classification in children with CP.

In this study, we developed a single-camera AI-based video classification framework for clinically defined grasp patterns during standardized upper-extremity tasks. The framework integrates MediaPipe-based hand landmark extraction, SAM2-based hand segmentation, YOLOv9-based object localization, and a 3D DenseNet-121 classifier. Rather than defining a new clinical assessment framework, the proposed approach was anchored to the Quality of Upper Extremity Skills Test (QUEST), a standardized clinical measure widely used in pediatric rehabilitation.

**Figure 1:**
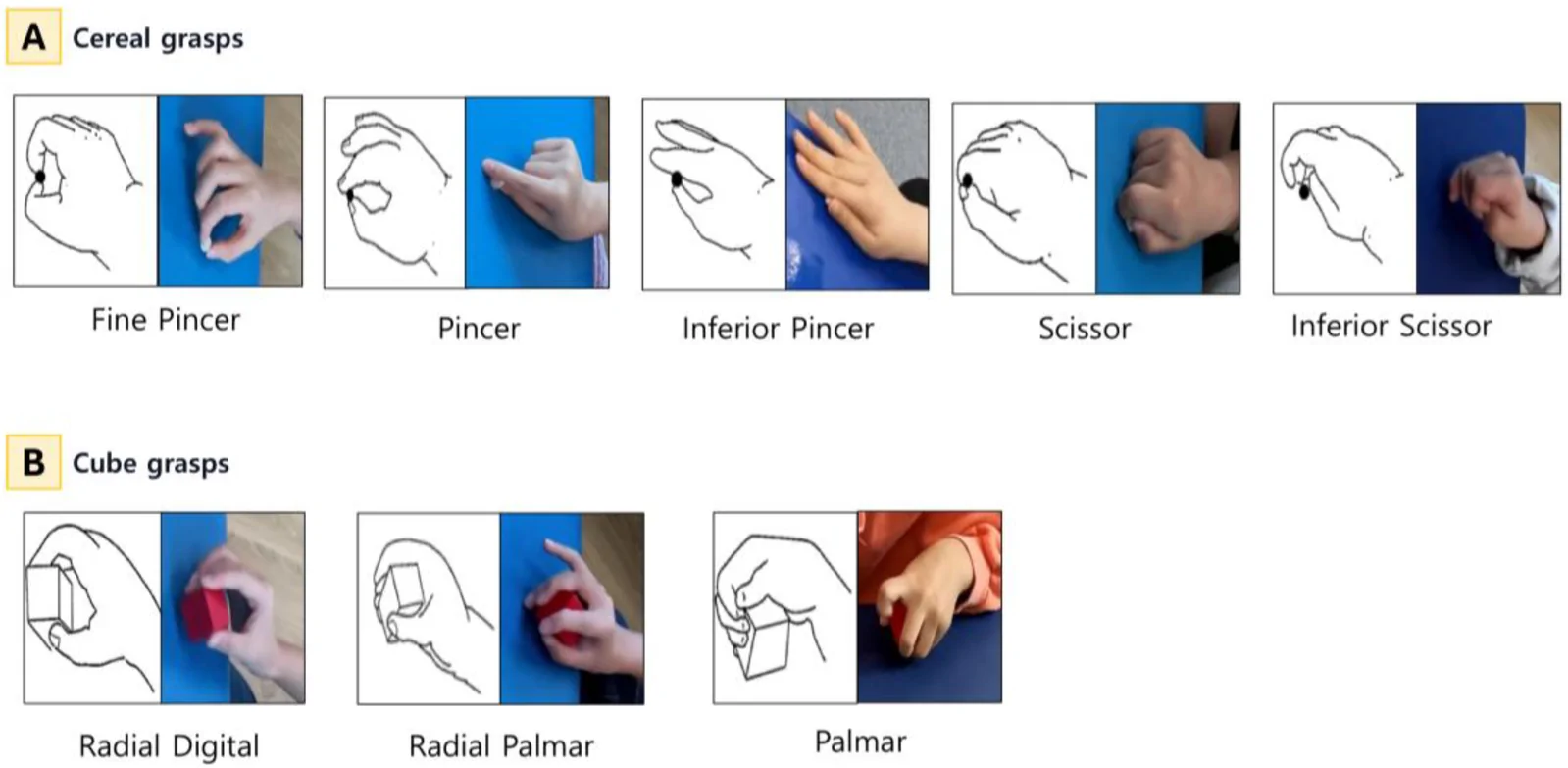
**Representative grasp patterns assessed in the Quality of Upper Extremity Skills Test (QUEST).** (**A**) Grasp-of-Cereal task illustrating five pincer-based grasp patterns: Fine Pincer, Pincer, Inferior Pincer, Scissor, and Inferior Scissor. (**B**) Grasp-of-Cube task illustrating three palmar-based grasp patterns: Radial Digital, Radial Palmar, and Palmar. Adapted from QUEST manual [8].

The QUEST Grasp-of-Cereal and Grasp-of-Cube tasks define developmentally ordered grasp categories based on thumb-finger coordination and hand–object interaction, thereby providing a clinically interpretable reference taxonomy for evaluating camera-derived movement information (Figure 1) [8]. We preserved the original QUEST grasp categories and evaluated whether the single-camera AI-based framework could classify these categories at the trial level. AI predictions were compared with expert occupational-therapist ratings, and ordinal performance metrics were used to account for the developmental distance between grasp categories.

The primary aim was to evaluate the feasibility and classification performance of a single-camera AI-based framework for clinically defined grasp-pattern recognition in children with CP. We hypothesized that the proposed framework would distinguish QUEST grasp categories with moderate-to-high accuracy while preserving clinically meaningful ordinal relationships between adjacent developmental levels. The intended role of the system is to support, rather than replace, standardized clinical assessment by providing an objective and reproducible video-based representation of hand function. Because earlier recordings contributed to model development and follow-up recordings acquired after the intervention were used for testing, some participants contributed data at both time points. The evaluation should therefore be interpreted as a longitudinal within-cohort proof of concept rather than as participant-independent validation in previously unseen children. The study was not designed to establish external generalizability or equivalence to clinical assessors.

## 2. Materials and Methods

### 2.1. Participants

This prospective cohort pilot study included children diagnosed with CP, aged 18 months to 8 years, who visited the pediatric rehabilitation clinic between September 2024 and August 2025. This study was conducted in accordance with the Declaration of Helsinki, and the protocol was approved by the Institutional Review Board of Asan Medical Center (No. 2023-0322). A total of 41 children contributed data to the study. Not all children completed both tasks: at the initial assessment, all 41 children performed the Cube task, whereas 37 performed the Cereal task; four children were unable to complete the Cereal task.

**Figure 2:**
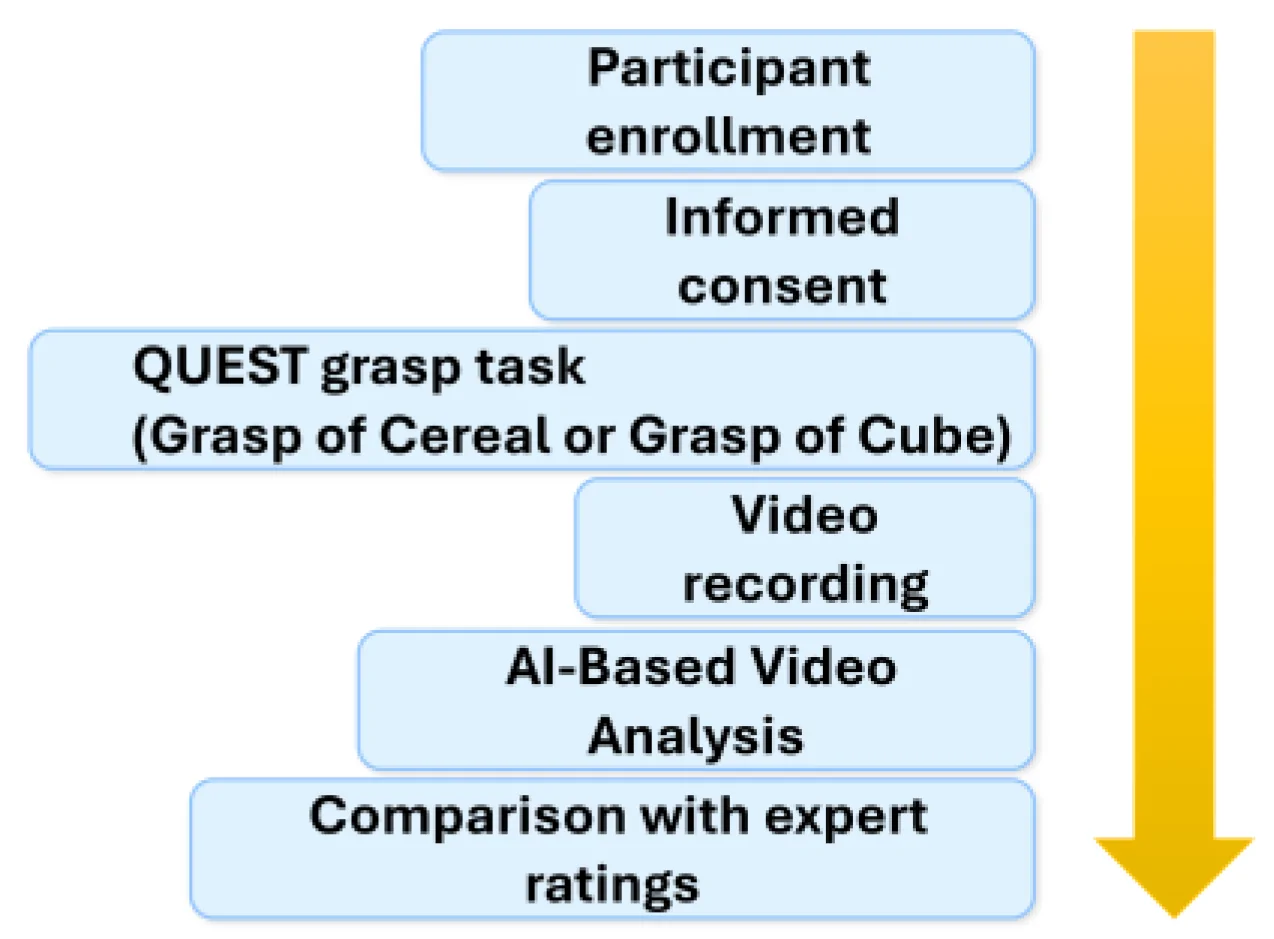
Flow diagram illustrating the study process from participant enrollment and informed consent through QUEST grasp tasks, video recording, AI-based analysis, and comparison with expert ratings. The yellow arrow indicates the sequential workflow.

Children were eligible if they were able to perform the QUEST grasp tasks and if video recording of hand movements was feasible. Exclusion criteria were as follows: (1) medical or behavioral conditions that precluded reliable video analysis, including severe visual obstruction or inability to engage with the task, (2) incomplete or unusable video recordings, and (3) refusal to provide written informed consent for study participation (Figure 2).

### 2.2. Clinical Information

The following clinical information was collected from medical records: age, sex, CP subtype (spastic unilateral, spastic bilateral, dyskinetic, or ataxic), and Manual Ability Classification System (MACS) level [9].

### 2.3. QUEST Assessment

The QUEST was used as the reference standard for grasp classification in children with CP. Only the grasp domain was analyzed, including the Grasp-of-Cereal and Grasp-of-Cube tasks.

For the Grasp-of-Cereal task, five developmentally ordered grasp categories were defined: Fine Pincer, Pincer, Inferior Pincer, Scissor, and Inferior Scissor. For the Grasp-of-Cube task, three grasp categories were defined: Radial Digital, Radial Palmar, and Palmar.

### 2.4. Ground-Truth Labeling

Ground-truth labels were assigned based on consensus between two experienced occupational therapists, who independently reviewed all recorded trials and resolved disagreements through discussion.

### 2.5. Video Recording of QUEST Performances

QUEST grasp performances were captured with a GoPro HERO10 Black RGB camera (GoPro Inc., San Mateo, CA, USA) at a resolution of 5.3K and 60 frames per second. The camera therefore served as the sole physical sensing device; all subsequent hand, object, and temporal features were derived computationally from the recorded RGB image sequence.

Each child was instructed to perform the task with both hands, and up to five trials were recorded per hand. When repeated trials were not feasible, at least one trial per hand was obtained. Each recorded trial was used as a trial-level analysis unit for classification; however, repeated trials from the same participant and hand are correlated observations and should not be interpreted as statistically independent samples. Recordings captured the task from initiation to completion under standardized conditions using a single frontal camera view. This fixed-view acquisition was selected to maintain a simple, clinically deployable sensing configuration, while recognizing that a single two-dimensional viewpoint can be affected by hand–object occlusion and loss of depth information.

### 2.6. Configuration of Training and Test Datasets

For both tasks, earlier pediatric recordings acquired at the first assessment contributed to model development, whereas follow-up recordings acquired after the intervention were used for pediatric testing. Table 1 summarizes the composition of the model-development dataset by data source (pediatric vs. adult) and the pediatric-only test dataset; it does not represent the internal training–validation split. Within the model-development dataset, pediatric participants were divided into training and validation subsets at an approximately 80:20 ratio at the participant level, ensuring that data from the same child were not distributed across those two internal subsets. Horizontal flipping was applied to pediatric examples in both the training and validation subsets to generate mirrored hand representations. This augmentation was used because some participants contributed recordings from only one hand; mirrored counterparts increased left–right hand variability and reduced dependence on a particular hand orientation. Adult demonstrations were recorded by one therapist and used only for model development; adult examples were likewise mirrored within the development data. After horizontal-flip augmentation, the combined development inventory comprised 3196 Cereal examples (1696 pediatric and 1500 adult; 53.1% and 46.9%, respectively) and 2460 Cube examples (1560 pediatric and 900 adult; 63.4% and 36.6%, respectively). Each adult category included 150 original and 150 mirrored examples. Table 2 reports absolute pediatric and adult counts by category. Inferior Pincer had no pediatric development examples. These counts describe 15-frame grasp examples after augmentation, not independent participants; mirrored examples do not constitute new independent observations. No pediatric-only comparison was performed, so whether adult supplementation improved pediatric classification remains unverified.

The pediatric dataset was derived from a longitudinal clinical program for children with cerebral palsy (CP), which included 8 weeks of fine motor training. The first-assessment recordings contributed to model development, and the second-assessment recordings obtained after the intervention constituted the test dataset. Consequently, some participants contributed recordings to both model-development and test datasets at different visits. For the Cube task, 39 of the 41 children contributing development data also contributed follow-up test recordings. Temporal separation does not provide participant-level independence because the same child’s hand morphology and individual movement characteristics may be represented at both time points. In addition, some test participants contributed multiple recording sessions and multiple trials from one or both hands. Participant identity was therefore retained in the evaluation records, and participant-level bootstrap resampling was used to account for within-child clustering in the available test records. The present evaluation should be interpreted as a longitudinal within-cohort proof of concept rather than as an estimate of generalization to previously unseen children.

### 2.7. Hand-Tracking Module Integrating MediaPipe Hands and SAM2

**Figure 3:**
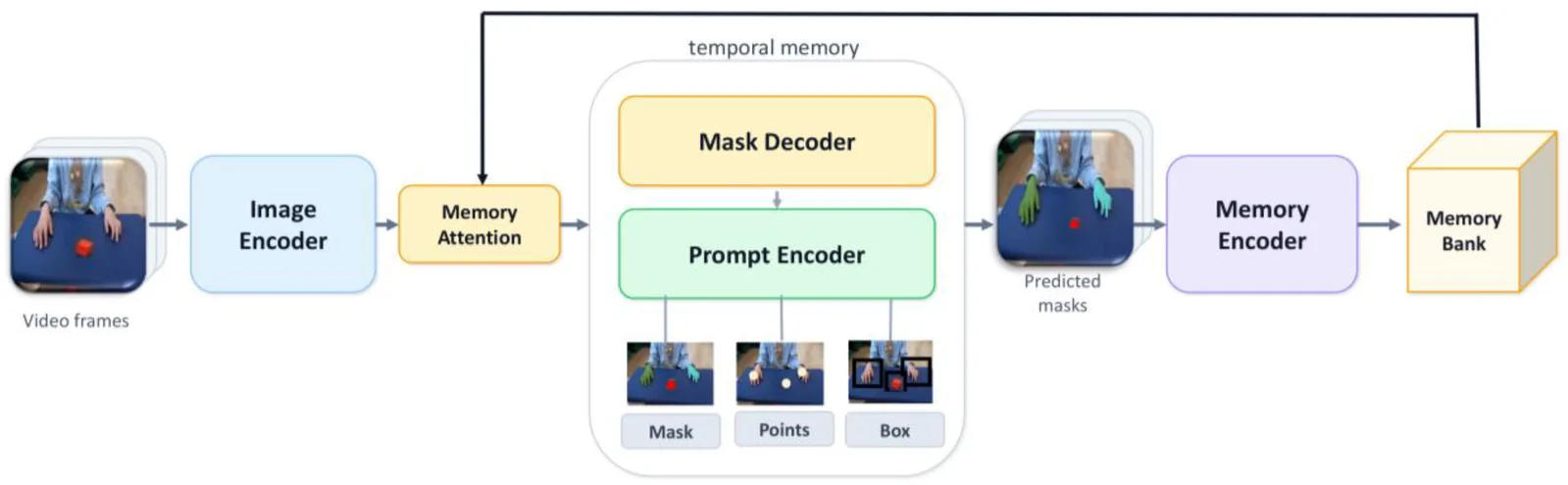
Overview of the hand-tracking module that combines MediaPipe Hands keypoints with the memory-based SAM2 video segmentation pipeline to produce temporally consistent hand masks. Arrows indicate the flow of image features, prompts, predicted masks, and temporal memory between processing stages. Colored overlays in the example frames depict segmentation masks; colored points and boxes indicate visual prompts.

Each RGB frame was first processed using MediaPipe Hands (version 0.10.14) [10] to extract 21 two-dimensional hand landmarks per detected hand (Figure 3). In parallel, the current frame was encoded by the SAM2 [11] image encoder. Prompts propagated from the preceding frame—including the hand mask, keypoint prompts, and anchor boxes—were provided to the SAM2 prompt encoder, and the mask decoder combined image and prompt features to estimate the hand mask for the current frame. The predicted mask was stored through the SAM2 memory mechanism and used to support temporal consistency in subsequent frames. This frame-by-frame propagation was repeated sequentially until the last frame of the recorded task video had been processed, at which point the loop terminated. This combination of landmark and segmentation information was intended to stabilize hand localization during rapid movement, partial occlusion, or transient landmark-detection failure. The open-source sources were Google AI Edge MediaPipe Hands (https://github.com/google-ai-edge/mediapipe (accessed on 20 October 2026)) and Meta SAM 2 (https://github.com/facebookresearch/sam2 (accessed on 20 October 2026); its repository setup.py identifies package version 1.0).

### 2.8. Hand Tracking and Preprocessing Pipeline

**Figure 4:**
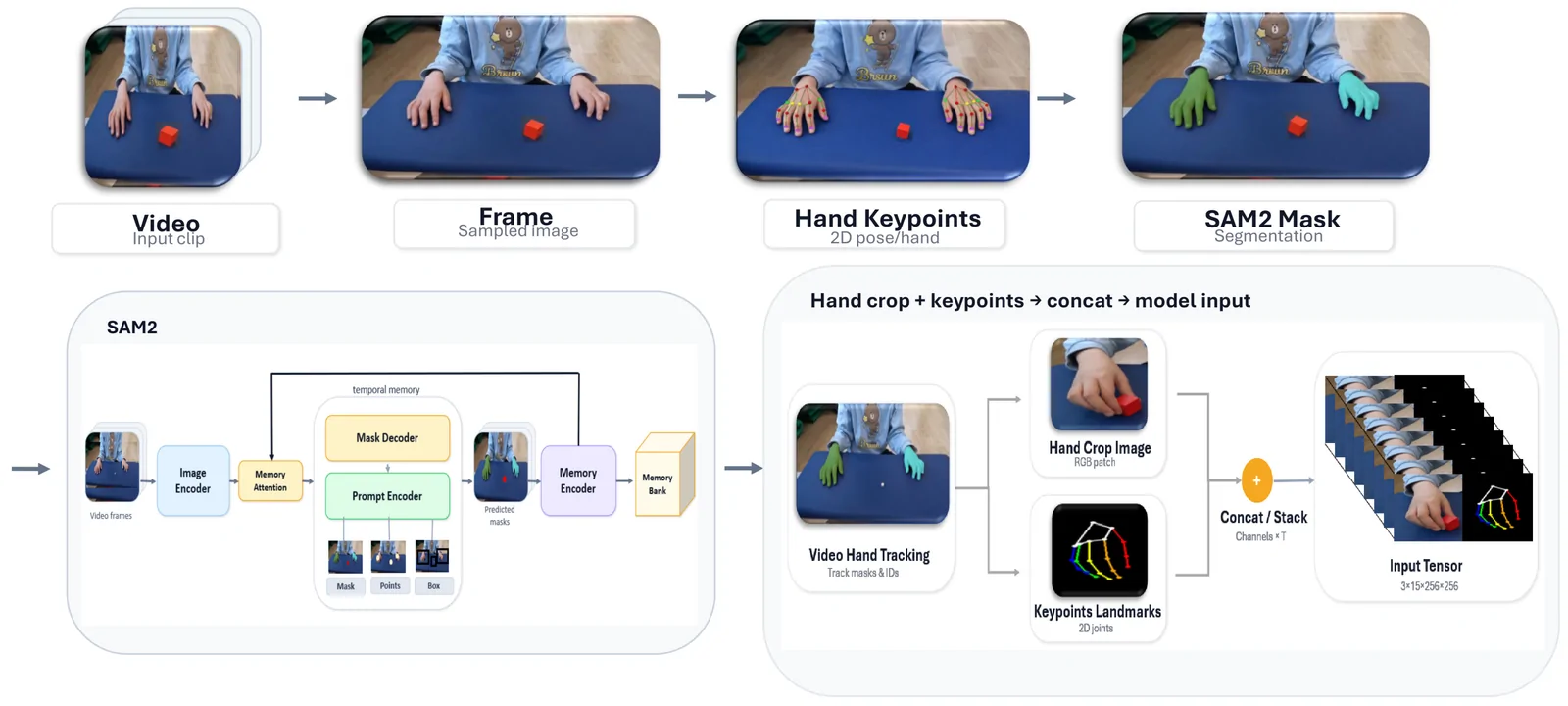
Preprocessing pipeline for hand-centric spatiotemporal input construction, including frame sampling, keypoint extraction, SAM2-based masking, hand-region cropping, and landmark visualization. Arrows indicate the processing sequence; colored hand overlays represent segmentation masks, and the landmark image visualizes detected hand keypoints.

As shown in Figure 4, recorded videos were resampled at a constant 30 frames per second for analysis. For each frame, MediaPipe Hands extracted 21 landmarks per detected hand; when both hands were visible, up to 42 landmarks and their associated confidence information were retained. SAM2 generated frame-wise hand masks, from which each hand region was isolated and resized to 256 × 256 pixels. A corresponding landmark visualization was generated by rendering the detected keypoints on a black background. For each frame, the RGB hand crop and the color landmark visualization were concatenated horizontally and the resulting composite image was resized to 256 × 256 pixels. The three channels represent the RGB channels of this composite image. Fifteen composite frames were stacked along the temporal dimension to form the 3 × 15 × 256 × 256 input tensor. The 15-frame windows were generated with a stride of five frames, giving a 10-frame overlap between consecutive windows. For example, a continuous segment of 45 frames produces seven full-length windows with this setting, compared with three non-overlapping windows.

### 2.9. 3D DenseNet-121–Based Hand Motion Classification

**Figure 5:**
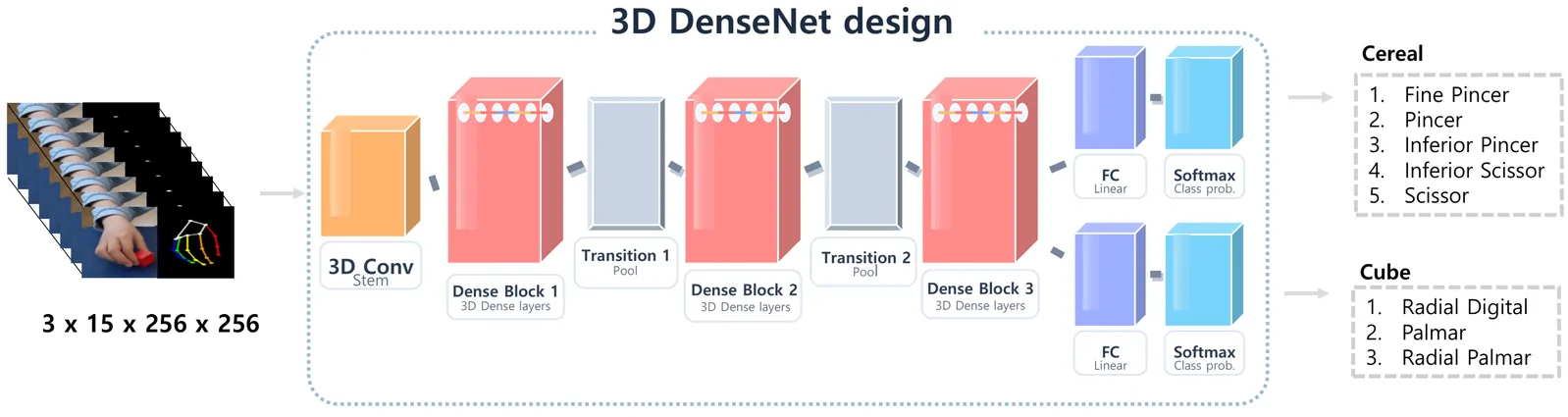
Architecture of the 3D DenseNet-121 model used for spatiotemporal hand motion classification from 15-frame hand clips. Arrows indicate the progression through convolutional, dense, transition, and task-specific classification layers; the colored blocks distinguish network components.

Figure 5 shows the 3D DenseNet-121 architecture used to classify grasp patterns from the camera-derived spatiotemporal representation. Each input consisted of a 15-frame, 3-channel tensor (3 × 15 × 256 × 256) combining segmented hand-region appearance and landmark-derived information. Three-dimensional convolution and pooling were used to extract spatial and temporal features, followed by Dense Blocks with feature reuse across layers [12,13]. The resulting representation was aggregated by global average pooling and mapped to the task-specific grasp categories through a fully connected output layer. The classifier used a 3D DenseNet-121 implementation from xmuyzz/3D-CNN-PyTorch (https://github.com/xmuyzz/3D-CNN-PyTorch) (accessed on 20 October 2026), with an input tensor of 3 × 15 × 256 × 256. The four dense blocks contained 6, 12, 24, and 16 layers. The growth rate was 32, the initial number of feature maps was 64, the bottleneck factor was 4, and transition layers reduced the number of channels by a factor of 0.5. The first convolution used a 3 × 7 × 7 kernel with a stride of (1, 2, 2). Convolutional weights were initialized using Kaiming normal initialization, and the model was trained from scratch. The output layer contained five classes for Cereal and three for Cube. Training used Adam (β1 = 0.9, β2 = 0.999), an initial learning rate of 1 × 10^−4^, a batch size of 8, and cross-entropy loss. Training was limited to 100 epochs. ReduceLROnPlateau reduced the learning rate by a factor of 0.1 after five epochs without improvement in validation loss. Early stopping used validation loss with a patience of 10 epochs, and the checkpoint with the lowest validation loss was selected for evaluation. Horizontal flipping was applied across the development dataset to generate mirrored counterparts and increase left–right hand variability, particularly for participants with recordings available from only one hand. A random seed of 42 was used. Training was conducted using Python 3.12.12, PyTorch 2.8.0+cu126, and the CUDA 12.6 build of PyTorch on an NVIDIA RTX 5090 GPU. The training configuration was selected empirically for this limited dataset based on stable training behavior observed in preliminary internal tests. This was not an exhaustive hyperparameter optimization, and the final test data were not used to select or adjust the training settings. The upstream DenseNet repository documents Python 3.8.5 and PyTorch 1.11.0 as its reference development environment; these source-repository versions are distinct from the training runtime reported here.

The 3D convolutional formulation was selected to model short-duration changes in hand configuration around object interaction rather than relying on a single static frame. Dense connectivity was used to promote feature reuse in the limited-data setting. The target classes were the five ordered Cereal grasp categories (Fine Pincer, Pincer, Inferior Pincer, Scissor, and Inferior Scissor) and the three Cube categories (Radial Digital, Radial Palmar, and Palmar).

### 2.10. Efficient Object Localization Using YOLOv9

YOLOv9 was trained from scratch without pretrained weights; no fine-tuning of a pretrained detector was performed. YOLOv9 [14] was used to localize the task object in the RGB video and provide frame-wise bounding boxes. The center of each detected bounding box was used as the object-position coordinate for the hand–object proximity calculation. These object coordinates were combined with the hand landmarks to identify the interaction period. For the Cereal task, the distance between the object center and index fingertip was compared with the frame-diagonal-normalized threshold, T = 0.04D, as described in the following subsection. An interaction trigger was then used to extract a 15-frame hand-centered segment for the 3D DenseNet-121 classifier. Thus, YOLOv9 supplied object-location information for temporal selection, while the DenseNet classifier assigned the QUEST grasp category from the selected hand segment. This interaction-triggered design focused classification on the grasp period rather than the entire task video. The YOLOv9 source code was obtained from https://github.com/WongKinYiu/yolov9 (accessed on 20 October 2026); its published requirements.txt specifies PyTorch >= 1.7.0 and torchvision >= 0.8.1 as minimum dependencies.

### 2.11. Hand–Object Interaction Detection and Temporal Cropping

**Figure 6:**
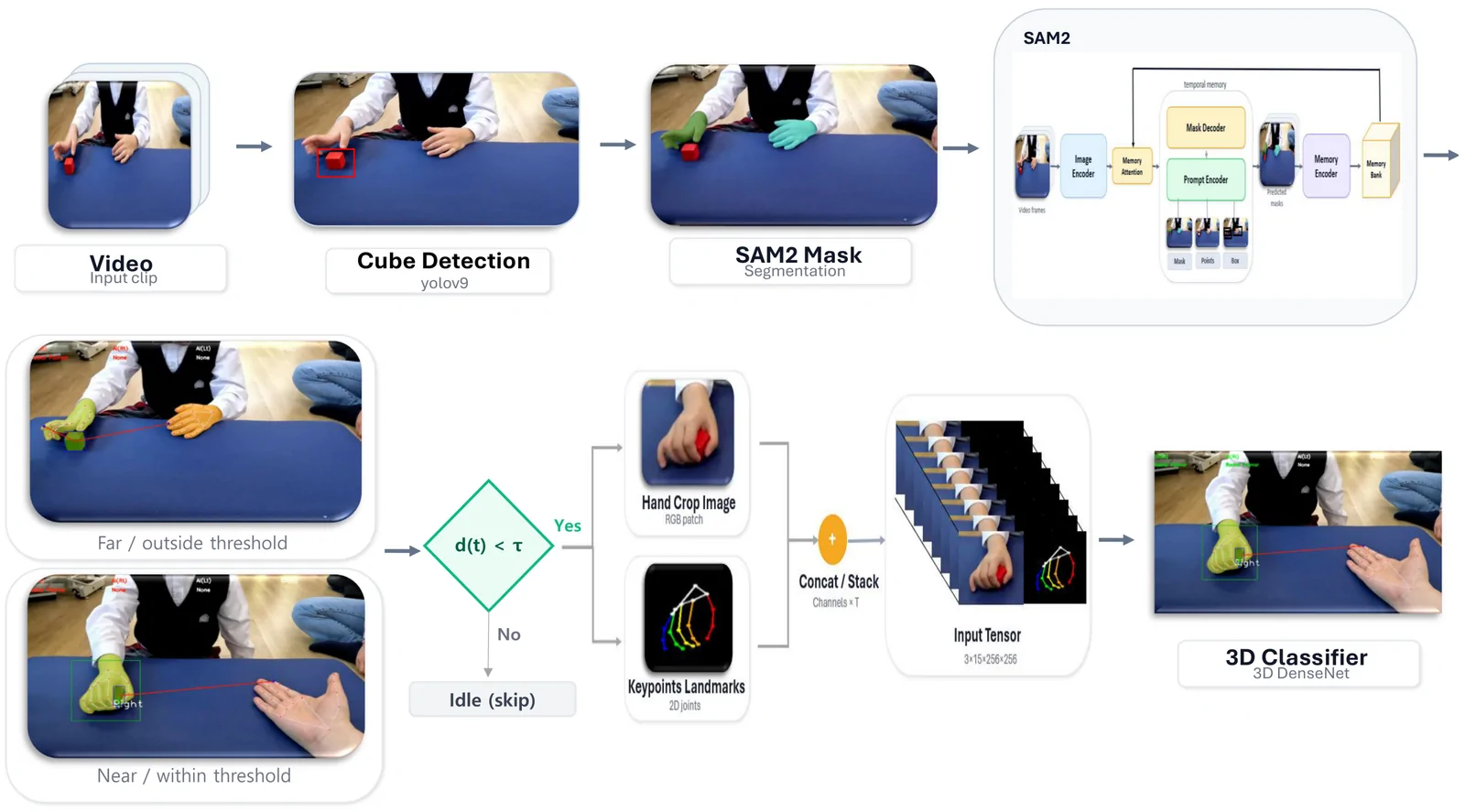
YOLOv9- and SAM2-based interaction-triggered motion analysis pipeline. Arrows indicate processing flow; the decision diamond represents the hand–object distance threshold, with the Yes branch triggering classification and the No branch skipping the frame. Colored hand overlays indicate segmentation masks, and red boxes show detected task objects.

Hand–object interaction was identified by combining object localization, hand-landmark tracking, and a distance-based trigger (Figure 6). For the Cereal task, YOLOv9 detected the cereal object, while MediaPipe and SAM2 provided fingertip coordinates and hand segmentation. In each frame, the Euclidean distance d between the detected object center (x_1_, y_1_) and index fingertip (x_2_, y_2_) was calculated using Equation (1):

*d* = √[(*x*_1_ − *x*_2_)^2^ + (*y*_1_ − *y*_2_)^2^]. (1)

To determine whether the hand is sufficiently close to the cereal, we define a dynamic distance threshold T based on the diagonal length D of the video frame with width W and height H, as given in Equation (2):

*D* = √(*W*^2^ + *H*^2^), *T* = 0.04 × *D*. (2)

An interaction event was triggered when d < T. The coefficient was selected using training recordings only. An experienced occupational therapist visually reviewed the training recordings and identified frames representing sufficient hand–object proximity for grasp interaction. The corresponding hand–object distances were measured in pixels, normalized by the frame diagonal D, and used to determine the fixed coefficient of approximately 0.04 in T = 0.04D. No final test recordings were used to determine or adjust this threshold. Thus, the threshold should be interpreted as a clinician-informed proximity criterion rather than a validated measure of physical contact. No threshold-sensitivity analysis was performed; consequently, robustness to other threshold values has not been established. The same fixed-threshold concept was used within the pipeline, but an index-fingertip proximity criterion may be less representative of palmar grasping in the Cube task.

During periods when the hand–object distance was below the interaction threshold, 15-frame windows were assembled with a stride of five frames and supplied to the classifier. Window predictions were recorded when a new window was inferred; recording stopped when the hand–object distance exceeded the threshold. Each trial was identified by task, participant, hand, and its start–end frame interval. The final trial-level prediction was determined from the first three available window-level predictions in temporal order using majority voting. When fewer than three predictions were available, all available predictions were used. If two or more classes received the same highest number of votes, the class appearing earliest among the selected predictions was assigned as the final trial-level class. The trial prediction was fixed before comparison with the therapist reference label; reference labels were not used to select a prediction. The final confusion matrices and performance metrics were computed from these trial-level predictions. Cereal records contained 1–38 window predictions per analyzed trial (median 6); 180 trials used three predictions, six used two, and six used one. Cube records contained 1–48 predictions (median 6); 371 trials used three, 16 used two, and nine used one. Twelve Cereal records and six Cube records had no stored prediction and were excluded from the analyzable-trial metrics. Five further Cube records without a valid reference category and one duplicate record were excluded. These exclusions mean that performance is conditional on the retained analyzable records; they do not establish complete pipeline success rates.

### 2.12. Statistical Analysis

Model evaluation was performed at the trial level using the pediatric test recordings. Confusion matrices compared trial-level predictions with therapist reference labels. Accuracy was the proportion of correctly classified trials. For K ordered classes indexed i = 0, …, K − 1, severity-weighted accuracy (SWA) was the mean of w_ij_ = 1 − |i − j|/(K − 1). Quadratic weighted kappa (QWK) used squared ordinal distances and chance correction. Cereal classes were ordered as follows: Fine Pincer, Pincer, Inferior Pincer, Scissor, and Inferior Scissor; Cube classes were ordered as follows: Radial Digital, Radial Palmar, and Palmar. This clinical order was applied consistently to reference and predicted labels. Class-wise precision, recall, and F1-score were calculated from the final confusion matrices. Macro-F1 and balanced accuracy were averaged over classes present in the reference labels; the absent Cereal Scissor reference class was excluded from these averages. For transparency, the five-class Cereal macro-F1 was also reported. Undefined precision for a class with no predictions was assigned zero, while recall for a class with no reference observations was not estimated.

Uncertainty in accuracy, SWA, and QWK was estimated using participant-level bootstrap resampling with 10,000 replicates and random seed 42. For each task, the observed number of children was sampled with replacement, and all retained trials from each sampled child, including both hands, were included together. Metrics were recalculated from the pooled confusion matrix in each replicate; the 2.5th and 97.5th percentiles defined the 95% confidence interval (CI). The bootstrap populations were 20 children for Cereal and 39 for Cube. Bootstrap CIs account for repeated sessions and trials within test participants but do not, by themselves, establish external generalizability [15]. Mean participant accuracy was additionally calculated by giving each child equal weight. Therapist agreement was summarized from its existing numerical matrices; participant-level CIs for therapist agreement were not calculated because linked individual ratings were not available.

The same accuracy, SWA, and QWK metrics were applied to the independent ratings of the two occupational therapists as a human-reference benchmark for error structure and ordinal consistency. Importantly, these evaluations are not methodologically equivalent: the AI model is compared with the consensus therapist label, whereas therapist performance reflects inter-rater agreement between two individual raters. Accordingly, therapist results are presented as a descriptive clinical reference rather than evidence of statistical equivalence or non-inferiority.

### 2.13. Use of Artificial Intelligence Tools

Large language models were used to assist language editing and the preparation of analysis scripts. They were not used to generate model predictions or clinical reference labels. Performance metrics were calculated programmatically from the recorded predictions and labels.

## 3. Results

### 3.1. Study Population and Cohort Composition

The Grasp-of-Cereal and Grasp-of-Cube tasks were analyzed separately within a cohort of 41 children. At the initial assessment, all 41 children performed Cube and 37 performed Cereal; four were unable to complete Cereal. Earlier recordings contributed to model development and follow-up recordings acquired after the intervention contributed to testing. The retained prediction records used for the Cereal performance and participant-bootstrap analyses represented 20 children and 192 trials; these 20 children constitute the evaluated Cereal test subset reported in Table 3. For Cube, 39 of the 41 children contributing development data also provided follow-up test recordings, and the Cube analysis included 396 trials from 39 children after exclusion of one duplicate record. Some test participants contributed multiple sessions and/or multiple trials. Evaluation keys therefore included participant ID to prevent records from different children from being combined and to support participant-level clustered resampling. Baseline characteristics of the development and follow-up cohorts are summarized in Table 3 and Table 4. Their sample sizes are participant counts, not numbers of statistically independent trials.

### 3.2. AI-Based Classification Performance for the Grasp-of-Cereal Task

**Figure 7:**
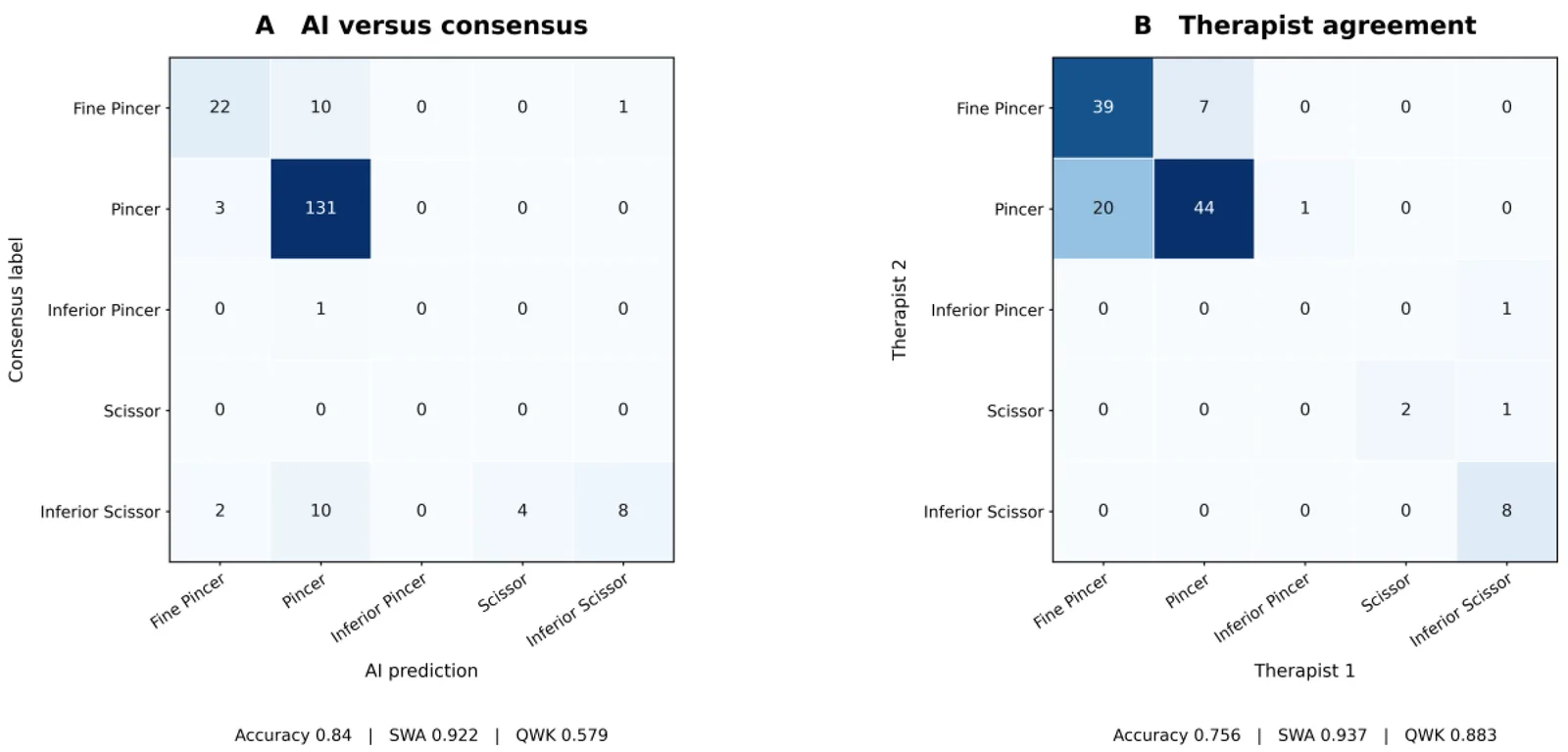
**Confusion matrices for AI-based and therapist-based classification of grasp patterns in the Grasp-of-Cereal task.** (**A**) Confusion matrix of AI-based predictions versus expert therapist labels. (**B**) Inter-rater agreement between two expert therapists. Overall accuracy, severity-weighted accuracy (SWA), and quadratic weighted kappa (QWK) are reported for each comparison. The AI matrix represents 192 trials from 20 children; the therapist matrix represents 123 independently rated trials. Darker blue cells indicate higher trial counts; rows show reference ratings and columns show comparison ratings.

The AI confusion matrix for 192 retained trials from 20 children is shown in Figure 7A. The model correctly classified 161 trials (accuracy, 0.84; participant-bootstrap 95% CI, 0.750–0.914). Class-wise performance was as follows: Fine Pincer, precision 0.815, recall 0.667, and F1-score 0.733 (33 reference trials); Pincer, precision 0.862, recall 0.978, and F1-score 0.916 (134 trials); Inferior Pincer, recall 0.000 with no predictions assigned to this class (1 trial); and Inferior Scissor, precision 0.889, recall 0.333, and F1-score 0.485 (24 trials). No Scissor reference trials were present, although four trials were predicted as Scissor. Macro-F1 across the four represented reference classes was 0.534 and balanced accuracy was 0.494; macro-F1 across all five classes, assigning zero to the absent class, was 0.427. Mean participant accuracy was 0.834. These results show that high overall accuracy was strongly influenced by the prevalent Pincer class. Table 5 and Table 6 summarize uncertainty and class-wise performance.

Cereal SWA was 0.922 (95% CI, 0.862–0.971) and QWK was 0.579 (95% CI, 0.281–0.855). Of the 31 errors, 18 (58.1%) were between adjacent classes and 13 (41.9%) crossed two or more stages. High SWA therefore coexisted with substantial non-adjacent errors and low balanced accuracy. The wide QWK interval indicates that ordinal agreement was imprecisely estimated in this small, imbalanced evaluation subset.

Therapist inter-rater agreement was calculated from 123 trials with independent ratings from both therapists, compared with 192 AI evaluation trials. The therapist matrix yielded accuracy 0.756, SWA 0.937, and QWK 0.883 (Figure 7B). AI-versus-consensus performance and therapist-versus-therapist agreement used different reference structures and subsets; these values do not provide a formal head-to-head comparison or establish equivalence.

### 3.3. AI-Based Classification Performance for the Grasp-of-Cube Task

**Figure 8:**
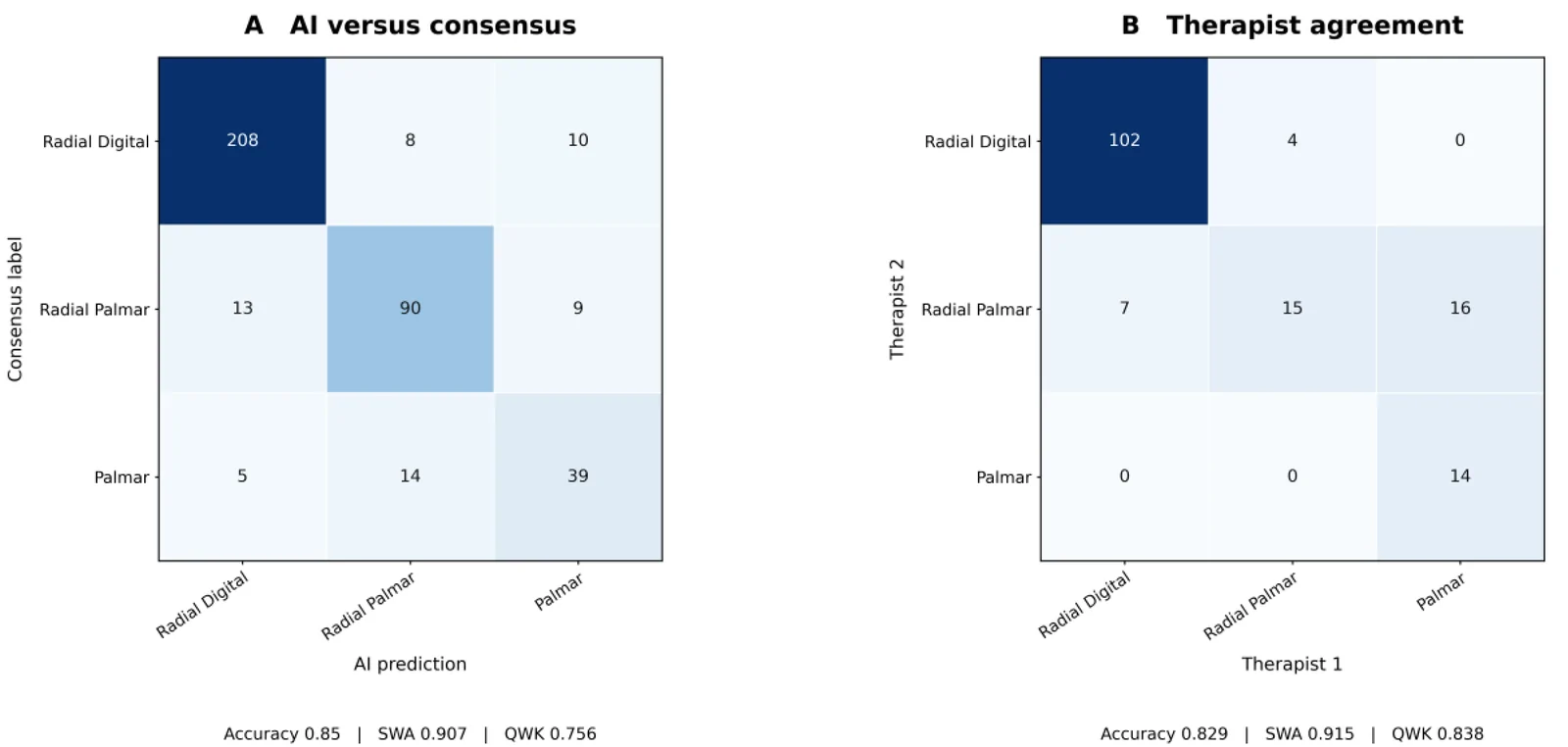
**Confusion matrices for AI-based and therapist-based classification of grasp patterns in the Grasp-of-Cube task.** (**A**) Confusion matrix of AI-based predictions versus expert therapist labels. (**B**) Inter-rater agreement between two expert therapists. Overall accuracy, SWA, and QWK are reported for each comparison. The AI matrix represents 396 trials from 39 children; the therapist matrix represents 158 independently rated trials. QWK uses the clinical order Radial Digital, Radial Palmar, and Palmar. Darker blue cells indicate higher trial counts; rows show reference ratings and columns show comparison ratings.

The confusion matrix for AI-based classification of the Grasp-of-Cube task is presented in Figure 8A. The model correctly classified 337 of 396 trials (accuracy, 0.85; participant-bootstrap 95% CI, 0.795–0.901). Class-wise precision, recall, and F1-score were 0.920/0.920/0.920 for Radial Digital (226 reference trials), 0.804/0.804/0.804 for Radial Palmar (112 trials), and 0.672/0.672/0.672 for Palmar (58 trials). Macro-F1 and balanced accuracy were both 0.799. Palmar had the lowest recall (39/58, 0.672). Errors occurred across adjacent categories, including 13 Radial Palmar trials classified as Radial Digital and 14 Palmar trials classified as Radial Palmar, with additional non-adjacent Radial Digital-Palmar errors.

For Cube, SWA was 0.907 (95% CI, 0.870–0.939) and QWK was 0.756 (95% CI, 0.636–0.849), using the clinical order Radial Digital, Radial Palmar, and Palmar. Mean participant accuracy was 0.847. Of the 59 errors, 44 (74.6%) were adjacent and 15 (25.4%) crossed two stages. Therapist agreement was evaluated on 158 independently rated trials and yielded accuracy 0.829, SWA 0.915, and QWK 0.838 (Figure 8B). The corrected model and therapist QWK values remain descriptive references because their labels and evaluation subsets differ.

## 4. Discussion

### 4.1. Overview of Findings

This study evaluated a single-camera AI-based classification framework that combines RGB recordings, hand landmark tracking, segmentation, object localization, and deep learning to classify clinically defined QUEST grasp patterns in children with CP. Trial-level accuracies were 0.84 and 0.85 for the Cereal and Cube tasks, respectively, supporting proof-of-concept feasibility. However, model-development and test recordings were temporally separated rather than fully participant-independent, with substantial overlap documented for the Cube task. Some test participants also contributed repeated sessions and multiple trials, and class-wise analysis revealed substantial imbalance-related heterogeneity; QWK values were lower than therapist inter-rater agreement. Accordingly, the present results should be interpreted as longitudinal within-cohort performance rather than as evidence of generalization to previously unseen children.

### 4.2. Interpretation of AI Performance

For the Grasp-of-Cereal task, the AI model achieved an accuracy of 0.84, an SWA of 0.922, and a QWK of 0.579, but balanced accuracy was 0.494 and macro-F1 across classes represented in the reference labels was 0.534. The prevalent Pincer class showed high recall (0.978), whereas Inferior Pincer had only one reference trial and was never predicted, and Inferior Scissor recall was 0.333. These class-level results clarify why high raw accuracy should be interpreted cautiously. Inferior Scissor was particularly challenging because the object may move beneath the thumb and become obscured in a frontal camera view. Adult demonstrations accounted for 46.9% of the augmented Cereal development examples, and the absence of pediatric Inferior Pincer training examples limits confidence in pediatric generalizability for that class. The favorable overall accuracy was therefore driven largely by the prevalent classes and does not establish reliable classification across the full QUEST grasp taxonomy. In particular, the present results do not support dependable clinical use for Inferior Pincer or Inferior Scissor. The single Inferior Pincer test trial is also insufficient to estimate performance precisely for that category.

For Cube, accuracy was 0.85, SWA was 0.907, and QWK was 0.756 using the clinical class order (Radial Digital, Radial Palmar, and Palmar). Macro-F1 and balanced accuracy were 0.799. Radial Digital had the highest recall (0.920), whereas Palmar had the lowest (0.672). The model QWK CI (0.636–0.849) reflects uncertainty within the evaluated cohort and does not support a formal comparison with therapist agreement. Rapid task completion, anterior–posterior cube movement, partial hand occlusion, and uncertainty in localizing initial contact may degrade the camera-derived hand–object representation. These possible mechanisms were not isolated by component-level experiments.

From a sensing perspective, these errors reflect structural limitations of single-view two-dimensional imaging. Landmark-based representations can reduce dependence on absolute hand size and appearance, but camera-based sensing remains vulnerable to self-occlusion, loss of depth information, and viewpoint variation. The combined use of RGB crops and landmark representations was intended to provide complementary visual and geometric information; nevertheless, the present results show that preprocessing robustness is critical when the target classes depend on subtle thumb-finger and hand–object relationships.

The model and therapists both showed confusion between neighboring grasp categories, but the two evaluations should not be treated as methodologically equivalent. AI predictions were judged against consensus therapist labels, whereas the human-reference matrices quantify agreement between individual therapists. The higher therapist QWK indicates more consistent ordinal agreement, and the current system should therefore be viewed as a proof-of-concept single-camera classification framework rather than as demonstrating therapist-equivalent performance or replacing therapist-administered QUEST assessment.

The sequential architecture of the sensing pipeline also creates opportunities for error propagation. The interaction-trigger threshold determines the start of the 15-frame analysis window; an early trigger can introduce pre-contact frames with limited grasp information, whereas a late trigger can omit the initial stable contact period. In addition, frame-to-frame instability in landmarks or segmentation masks can produce inconsistent hand crops and degrade the spatiotemporal representation supplied to the 3D network. Future implementations could improve robustness through adaptive interaction detection, temporal smoothing of landmarks and masks, confidence-aware frame weighting, or end-to-end approaches that jointly optimize temporal localization and classification.

### 4.3. Comparison with Previous Studies

Previous AI-based studies of pediatric hand or motor function have often focused on binary classification or task-level outcomes [3,4,16]. Ye et al. used video analysis to derive task duration, attempts, and successful trials during developmental testing, while Hwang et al. analyzed features of children’s drawings to estimate fine-motor maturity. Reich et al. used pose-based joint tracking for binary classification of infant movement patterns. In contrast, the present study uses camera-derived hand and object information to classify multiple developmentally ordered, clinically defined grasp patterns within an established clinical assessment. This formulation is clinically interpretable because the output categories correspond directly to QUEST grasp definitions rather than to latent model features alone. It also provides an engineering framework for studying how a functional grasp is performed, not merely whether a task is completed.

Recent markerless validation studies also underline the distinction between technical feasibility and clinical validation. Upper-limb kinematic agreement with an optoelectronic reference [5] does not establish correctness of QUEST grasp labels, while stroke-rehabilitation [6] and ergonomic [7] evidence concerns different populations and tasks. Pediatric grasp classification requires its own validation, particularly for occluded and underrepresented patterns.

### 4.4. Limitations

Several limitations should be acknowledged. First, the pediatric dataset was small and the test classes were markedly imbalanced. Adult demonstrations accounted for 46.9% of augmented Cereal development examples and 36.6% of augmented Cube examples; Inferior Pincer had no pediatric development examples. Adult and pediatric hands may differ in morphology, movement speed, grasp strategy, and movement variability. No pediatric-only training comparison was performed, so neither the benefit nor the cost of adult supplementation can be established. Domain adaptation is one possible future approach to distribution shift [17], but it was not implemented or evaluated in this study. Augmented sample counts should not be interpreted as numbers of independent observations.

Second, participant-level independence between model-development and test datasets was incomplete. The first-assessment recordings contributed to model development, whereas follow-up recordings acquired after the intervention were used for testing; for the Cube task, 39 of the 41 children contributing development data also contributed follow-up test recordings. Temporal separation does not eliminate potential participant-specific visual or movement information, including hand morphology and individual movement characteristics, and the post-intervention test distribution may also differ systematically from the earlier recordings. In addition, some test participants contributed multiple sessions, multiple trials, and data from one or both hands, so these observations are correlated. Participant-level bootstrap CIs were calculated by resampling children and retaining all available sessions, hands, and trials from each selected child together. This approach addresses within-child clustering in the available evaluation records but cannot correct development-test participant overlap, selection of analyzable records, or small class-specific sample sizes. The broad Cereal QWK CI (0.281–0.855) indicates substantial uncertainty in ordinal agreement. The 192 Cereal and 396 Cube trials should not be interpreted as equivalent numbers of independent participants. External validation using strict participant-level separation remains necessary to establish generalizability to unseen children [15].

Third, all recordings were acquired under controlled conditions using a single frontal RGB camera. This standardized setup supports reproducibility but does not establish robustness to different camera positions, lighting conditions, backgrounds, devices, or unconstrained clinical and home environments.

Fourth, no component-wise ablation, threshold-sensitivity analysis, or quantitative component-failure analysis was performed. The threshold was derived from clinician-selected proximity frames in training recordings only, without using the final test set. Nevertheless, the stability of classification across threshold values is unknown. The independent contribution of MediaPipe, SAM2, YOLOv9, and the 3D classifier cannot be isolated from the current results. The present study demonstrates the feasibility of the integrated pipeline, rather than the validated robustness or independent superiority of its components. Future evaluations should distinguish sensing and preprocessing failures from classifier errors and assess error propagation.

### 4.5. Future Directions

Future studies should prioritize larger pediatric datasets with adequate representation of all grasp categories and strict participant-level separation across training, validation, and test sets. External validation across camera devices and clinical environments will be important for establishing generalizability. Age- and hand-size-related variability should also be examined explicitly rather than being addressed primarily through adult demonstration data.

Methodologically, adaptive temporal localization may better capture short, information-rich grasp segments than fixed-window vote-based aggregation. Multi-view or depth sensing could reduce occlusion and recover three-dimensional hand–object relationships, while confidence-aware fusion of RGB, landmarks, segmentation, and object trajectories may improve robustness. These developments would further align the system with the broader goal of intelligent biomedical sensing for quantitative movement assessment.

With these improvements, single-camera AI-based grasp classification could support objective and reproducible monitoring of hand function while complementing, rather than replacing, standardized therapist assessment. The present framework also illustrates how vision-based sensing and machine learning can translate clinically defined movement categories into quantitative digital outputs for future rehabilitation monitoring and human–machine interaction applications.

## 5. Conclusions

The integrated single-camera pipeline demonstrated longitudinal within-cohort classification of QUEST grasp patterns in children with CP. Accuracies of 0.84–0.85 should be interpreted alongside the Cereal macro-F1 of 0.534 and balanced accuracy of 0.494, which indicate uneven class performance. Participant-level bootstrap quantified uncertainty while accounting for repeated sessions and trials within test participants. Nevertheless, incomplete participant-level independence between model development and testing, limited pediatric examples, selection of analyzable recordings, and unverified effects of adult supplementation restrict generalization. The findings do not establish dependable classification of all grasp categories, clinical equivalence to therapists, or performance in previously unseen children. Larger participant-independent external validation remains necessary before clinical application.

## Figures and Tables

**Table 1:** Composition of development and test datasets by data source.

Task	Development: Pediatric	Development: Adult	Test: Pediatric
Grasp of Cereal	53.1% (1696/3196)	46.9% (1500/3196)	100% pediatric test data
Grasp of Cube	63.4% (1560/2460)	36.6% (900/2460)	100% pediatric test data

**Table 2:** Class-wise grasp counts before and after horizontal-flip augmentation.

Task	Grasp Category	Pediatric Original (*n*)	Pediatric After Augmentation (*n*)	Adult After Augmentation (*n*)	Total After Augmentation (*n*)
Cereal	Fine Pincer	209	418	300	718
	Pincer	221	442	300	742
	Inferior Pincer	0	0	300	300
	Scissor	207	414	300	714
	Inferior Scissor	211	422	300	722
Cereal Total	Total	848	1696	1500	3196
Cube	Radial Digital	323	646	300	946
	Radial Palmar	243	486	300	786
	Palmar	214	428	300	728
Cube Total	Total	780	1560	900	2460

Counts in Table 2 refer to 15-frame grasp examples before and after horizontal-flip augmentation across the model-development dataset, pooling the training and validation subsets. For the pediatric data, the Original column reports examples before mirroring and the after-augmentation column reports the corresponding total after horizontal flipping. Horizontal flipping was applied in both the training and validation subsets; because each original pediatric example was mirrored once, the pediatric counts doubled (e.g., Cereal: 848 original to 1696 after augmentation; Cube: 780 original to 1560 after augmentation). The adult column reports post-augmentation counts; each adult category comprises 150 original and 150 mirrored examples (300 after augmentation). Totals are post-augmentation counts pooled across pediatric and adult development data. These counts are not numbers of unique children, independent physical trials, or YOLOv9 annotated detection images.

**Table 3:** Baseline characteristics of children in the development and test datasets for the Grasp-of-Cereal task.

Variables	Development Set (*n* = 37)	Test Set (*n* = 20)
Age (years)	5.41 ± 1.46	5.8 ± 1.01
Sex (male:female)	30 (81):7 (19)	15 (75):5 (25)
CP type		
Spastic, bilateral	13 (35)	6 (30)
Spastic, unilateral	22 (59)	12 (60)
Dyskinetic	1 (3)	1 (5)
Ataxic	1 (3)	1 (5)
MACS level (I:II:III:IV:V)	1 (3):15 (41):20 (54):1 (3):0 (0)	1 (5):10 (50):8 (40):1 (5):0 (0)

Values are presented as mean ± standard deviation or number (%), unless otherwise indicated. Percentages are rounded and may not total 100%. CP, cerebral palsy; MACS, Manual Ability Classification System. The test column describes the 20 children represented in the retained Cereal prediction records used for classification and participant-bootstrap analyses.

**Table 4:** Baseline characteristics of children in the development and test datasets for the Grasp-of-Cube task.

Variables	Development Set (*n* = 41)	Test Set (*n* = 39)
Age (years)	5.54 ± 1.40	5.62 ± 1.21
Sex (male:female)	32 (78):9 (22)	28 (72):11 (28)
CP type		
Spastic, bilateral	11 (27)	11 (28)
Spastic, unilateral	28 (68)	26 (67)
Dyskinetic	1 (2)	1 (3)
Ataxic	1 (2)	1 (3)
MACS level (I:II:III:IV:V)	1 (2):17 (42):22 (54):1 (2):0 (0)	1 (3):17 (44):19 (49):2 (5):0 (0)

Values are presented as mean ± standard deviation or number (%), unless otherwise indicated. Percentages are rounded and may not total 100%. CP, cerebral palsy; MACS, Manual Ability Classification System.

**Table 5:** Trial-level performance with participant-bootstrap 95% confidence intervals.

Task	Children	Trials	Accuracy (95% CI)	SWA (95% CI)	QWK (95% CI)
Cereal	20	192	0.839 (0.750–0.914)	0.922 (0.862–0.971)	0.579 (0.281–0.855)
Cube	39	396	0.851 (0.795–0.901)	0.907 (0.870–0.939)	0.756 (0.636–0.849)

CIs were obtained from 10,000 participant-level bootstrap replicates with seed 42; all retained sessions, both hands, and all retained trials from each selected child were resampled together. QWK used the clinical ordinal class order. These intervals account for clustering of repeated observations within test participants but do not remove development-test participant overlap, selection of analyzable records, small class-specific sample sizes, or limitations in external generalizability.

**Table 6:** Class-wise precision, recall, and F1-score.

Task	Reference Class	Support	Precision	Recall	F1
Cereal	Fine Pincer	33	0.815	0.667	0.733
	Pincer	134	0.862	0.978	0.916
	Inferior Pincer	1	0.000	0.000	0.000
	Scissor	0	0.000	NE	NE
	Inferior Scissor	24	0.889	0.333	0.485
Cube	Radial Digital	226	0.920	0.920	0.920
	Radial Palmar	112	0.804	0.804	0.804
	Palmar	58	0.672	0.672	0.672

NE, not estimated because the reference class was absent. Precision for classes with no predictions was set to zero. Cereal macro-F1 over four reference-present classes was 0.534 (all five classes: 0.427), and balanced accuracy was 0.494. Cube macro-F1 and balanced accuracy were 0.799.

## Data Availability

The datasets generated during the current study are not publicly available due to ethical and privacy considerations, but are available from the corresponding author upon reasonable request.
